# Perceived Stress, Type D Personality and Coping Strategies Among Hospitalized Oncology Patients: A Cross-Sectional Study

**DOI:** 10.3390/jcm15166213

**Published:** 2026-08-11

**Authors:** Robert Jan Łuczyk, Kamil Sikora, Agnieszka Zawada, Marta Łuczyk, Dorota Weber, Anna Charuta

**Affiliations:** 1 Institute of Health Sciences, Faculty of Medical and Health Sciences, University of Siedlce, 08-110 Siedlce, Poland; 2 Department of Internal Medicine and Internal Nursing, Faculty of Health Sciences, Medical University of Lublin, 20-093 Lublin, Poland; 3 Long-Term Care Nursing Department, Faculty of Health Sciences, Medical University of Lublin, 20-093 Lublin, Poland; 4 Faculty of Health Sciences, University of Economics and Innovation in Lublin, 20-209 Lublin, Poland

**Keywords:** stress, cancer, coping with stress, oncology patients, Type D personality, psycho-oncology

## Abstract

**Background:** Stress is regarded as one of the natural and unavoidable elements of human life and, at the same time, one of the key determinants of physical and mental health. Stress is not a new phenomenon; it has accompanied human beings since the beginning of life as a reaction to everyday challenges. This study aimed to assess the prevalence and severity of stress, as well as stress-coping strategies, in a group of patients treated for oncological reasons. **Methods:** The study included 108 patients hospitalized at the Independent Public Clinical Hospital No. 1 in Lublin, Poland, in the following departments: the 2nd Department of General, Gastroenterological and Gastrointestinal Oncological Surgery; the Department of Oncological Surgery; and the Department of Hemato-Oncology and Bone Marrow Transplantation. Three standardized questionnaires were used: the Perceived Stress Scale (PSS-10), the Type D Personality Scale (DS-14), and the “How Do I Cope” Inventory (JSR). Statistical analysis was performed with IBM SPSS Statistics, version 25; statistical significance was set at α < 0.05. **Results:** Patients treated for oncological reasons constitute a group exceptionally exposed to stress, which accompanies them at every stage of the disease, from diagnosis, through treatment, to the terminal phase. In patients whose treatment resulted in remission, stress related to the possibility of recurrence accompanies every follow-up examination and every worrying symptom. Given the range of situations that provoke stress, the overall level of stress in this group can be regarded as high. Stress-coping strategies among oncological patients are shaped by multiple factors, including selected sociodemographic characteristics; taking into account the diversity of these factors, coping strategies varied considerably across the sample. The level of coping strategies was not associated with age or sex but was significantly associated with educational attainment. **Conclusions:** Oncological patients experience a high and pervasive level of stress that persists throughout diagnosis, treatment, and follow-up. Stress-coping strategies are heterogeneous, are not determined by age or sex, but are shaped by education, indicating a need for individualized psychosocial and educational support integrated into routine oncological care.

## 1. Introduction

Cancer is a disease whose underlying mechanism involves dysregulation of the genes that control cell proliferation, ultimately leading to uncontrolled cell division. The development of cancer is influenced by multiple factors, including chemical, physical, viral, and bacterial agents, as well as hormonal medications [1].

It is essential to view oncological disease from two equally important perspectives: medical and psychological. The medical perspective focuses on the extensive damage that cancer causes to the human body and its normal functioning, whereas the psychological perspective concerns the impact of cancer on the patient’s social life, the emotions experienced by the patient, and the disruption of social roles resulting from disease progression [2].

Stress continuously affects our lives; we encounter it every day, in a variety of situations, and often find ourselves unable to cope with it effectively. The fast pace of life and ongoing civilizational change mean that exposure to stress is steadily increasing. A high level of stress diminishes life satisfaction, lowers self-esteem, and causes frustration, which in turn can contribute to the development of numerous conditions—not only psychological but also those affecting the physiological functioning of the body [3].

Cancer is often perceived as the disease associated with the greatest degree of stress. Patients encounter it from the moment of diagnosis, and it continues to accompany them at every stage of their struggle with the disease [4].

The ability to cope effectively with stress caused by cancer depends on individual predispositions, including personality traits and character, as well as the capacity for positive thinking and adjustment to illness. The better a patient’s adjustment—understood as acceptance of the current situation combined with a positive outlook on the future—the greater the likelihood that the patient will cope successfully with stress [5].

Most previous studies in psycho-oncology have examined perceived stress, personality traits, and coping strategies separately, even though these constructs are theoretically and empirically interconnected: personality characteristics such as Type D personality are known to shape both the cognitive appraisal of stressors and the choice of coping strategies, which in turn determine the level of psychological distress ultimately experienced by the patient [4,5,6]. A stress-prone personality profile combined with maladaptive coping may therefore identify patients at particularly high risk of chronic psychological distress, while this combination cannot be captured when the three constructs are assessed in isolation. Simultaneous assessment of perceived stress, Type D personality, and coping strategies may consequently provide a more complete picture of the psychological burden experienced by hospitalized oncology patients and support more precise targeting of psycho-oncological interventions. To date, however, this combined assessment approach has rarely been applied to hospitalized patients undergoing surgical or hemato-oncological treatment in Poland, which constitutes an important gap in the existing evidence that the present study seeks to address. Specifically, this study adds to the existing literature by jointly quantifying perceived stress, Type D personality, and coping strategies within a single, well-defined hospitalized cohort rather than relying on separate studies of each construct, and testing their associations with routinely available sociodemographic characteristics (sex, age, education, and place of residence), thereby identifying subgroups of oncology patients—such as those with lower educational attainment or residing outside large urban centers—who may benefit most from targeted psycho-oncological support.

Building on this rationale, and despite the well-documented psychological burden of cancer, data on the level of perceived stress and the coping strategies used by hospitalized oncology patients treated at surgical and hemato-oncological centers in Poland remain limited. The present study was therefore designed to assess the severity of stress and stress-coping strategies in this population and to examine their associations with sociodemographic characteristics.

## 2. Materials and Methods

### 2.1. Study Design and Participants

This cross-sectional, questionnaire-based (diagnostic survey) study included 108 patients hospitalized at the Independent Public Clinical Hospital No. 1 in Lublin, Poland, in the following departments: the 2nd Department of General, Gastroenterological and Gastrointestinal Oncological Surgery; the Department of Oncological Surgery; and the Department of Hemato-Oncology and Bone Marrow Transplantation. Data were collected between December 2021 and April 2025. Participation was anonymous and voluntary; after the purpose and scope of the study had been explained, informed consent was obtained from all participants.

Patients were recruited using consecutive sampling among individuals hospitalized in the three participating departments during the study period. Inclusion criteria were a confirmed oncological diagnosis, hospitalization in one of the participating departments, age ≥ 18 years, and the ability to understand and complete the questionnaires independently. Exclusion criteria were severe cognitive impairment or a clinical condition precluding questionnaire completion, and refusal to participate. A separate screening log was not maintained; therefore, the number of patients who declined participation could not be determined retrospectively.

### 2.2. Study Instruments

The Perceived Stress Scale (PSS-10) is the Polish adaptation, by Zygfryd Juczyński and Nina Ogińska-Bulik, of the Cohen Perceived Stress Scale. It is a 10-item tool that measures the intensity of stress experienced by an individual in relation to difficult events and problems, as well as the perceived ability to manage them [7,8].

The Type D Personality Scale (DS-14) is a 14-item instrument developed by Johan Denollet and adapted into Polish by Nina Ogińska-Bulik and Zygfryd Juczyński. It comprises two 7-item subscales: Negative Affectivity, which measures the tendency to experience negative emotions; and Social Inhibition, which measures the tendency to refrain from expressing these emotions and related behaviors. It is used to assess the stress-prone (Type D) personality profile in both healthy and chronically ill adults [8,9]. According to the original scoring procedure, Type D personality is conventionally classified when a respondent scores at least 10 points on both the Negative Affectivity and Social Inhibition subscales [9]. In the present study, the two DS-14 dimensions were analyzed as continuous scores; therefore, the prevalence of categorical Type D personality was not estimated.

The “How Do I Cope” Inventory (JSR) was likewise developed by Juczyński and Ogińska-Bulik and provides information on how a person behaves when confronted with a difficult situation, yielding three subscale scores: active coping, emotion-focused coping, and seeking social support [8].

### 2.3. Statistical Analysis

Statistical analyses were performed with IBM SPSS Statistics, version 25 (IBM Corp., Armonk, NY, USA). Statistical significance was set at α < 0.05. Descriptive statistics for the PSS-10, DS-14, and JSR questionnaires included the mean, standard deviation, median, mean rank, skewness, and kurtosis coefficients, together with the Kolmogorov–Smirnov test of normality. Sociodemographic characteristics were described using frequencies and percentages. Ninety-five percent confidence intervals for the mean and Student’s *t*-test were used to draw inferences about the level of stress, Type D personality, and coping strategies. Associations between stress level, Type D personality, coping strategies, and age were assessed with Spearman’s rho correlation coefficient. Associations between stress level, Type D personality, coping strategies, and sociodemographic variables were assessed with the Kruskal–Wallis H test, followed by post hoc pairwise comparisons where appropriate.

The choice between parametric and non-parametric procedures was guided by the specific objective of each analysis rather than applied uniformly across the study. Three distinct analytical aims were pursued. I. To characterize the distribution of each scale, descriptive statistics and the Kolmogorov–Smirnov test were computed; because this test indicated statistically significant deviations from normality for the PSS-10, DS-14, and all JSR subscales, non-parametric procedures were adopted for all subsequent within-sample analyses of these variables. II. To compare the mean level of stress, Type D personality traits, and coping strategies observed in the present sample with fixed, previously published normative reference values for the Polish adult population, one-sample Student’s *t*-tests were used. This comparison tests the a priori null hypothesis that the study-group mean equals a known, externally fixed population parameter (the published normative mean) rather than comparing two empirically observed samples; such one-sample comparisons against a numerically fixed reference value are commonly considered acceptably robust to moderate non-normality at the present sample size (*n* = 108), consistent with the Central Limit Theorem. III. To examine within-sample associations between the psychological variables and sociodemographic characteristics—for which the normality assumption did not hold; Spearman’s rho correlation coefficient was used for the continuous variable age, and the Kruskal–Wallis H test, followed by post hoc pairwise comparisons where appropriate, was used for the categorical sociodemographic variables (sex, education, place of residence). This distinction between normative-benchmark comparisons (parametric) and within-sample associations (non-parametric) determined the statistical test applied to each research question.

Ninety-five percent confidence intervals for the mean accompanied all normative-benchmark comparisons described above.

Results are presented in tables summarizing descriptive and inferential statistics. Descriptive and distributional statistics for the DS-14 and JSR subscales are consolidated in Table, while one representative histogram is retained to illustrate the distribution of the primary outcome, perceived stress.

## 3. Results

### 3.1. Characteristics of the Study Group

The study group comprised 108 oncology patients; their characteristics are summarized below. Nearly half of the participants were female (47.22%) and just over half were male (51.85%); one respondent (0.93%) did not answer this question. Regarding age, 13.89% of respondents were 18–25 years old, 15.74% were 26–35, 22.22% were 36–45, 17.59% were 46–55, 16.67% were 56–65, and 13.89% were older than 65 years. With respect to education, 7.41% of respondents had primary education, 17.59% vocational education, 38.89% secondary education, and 36.11% higher education. Regarding place of residence, 16.67% lived in a town of up to 100,000 inhabitants, 60.19% in a city of more than 100,000 inhabitants, and 23.15% in a rural area.

### 3.2. Prevalence of Stress Among Oncology Patients

**Figure 1:**
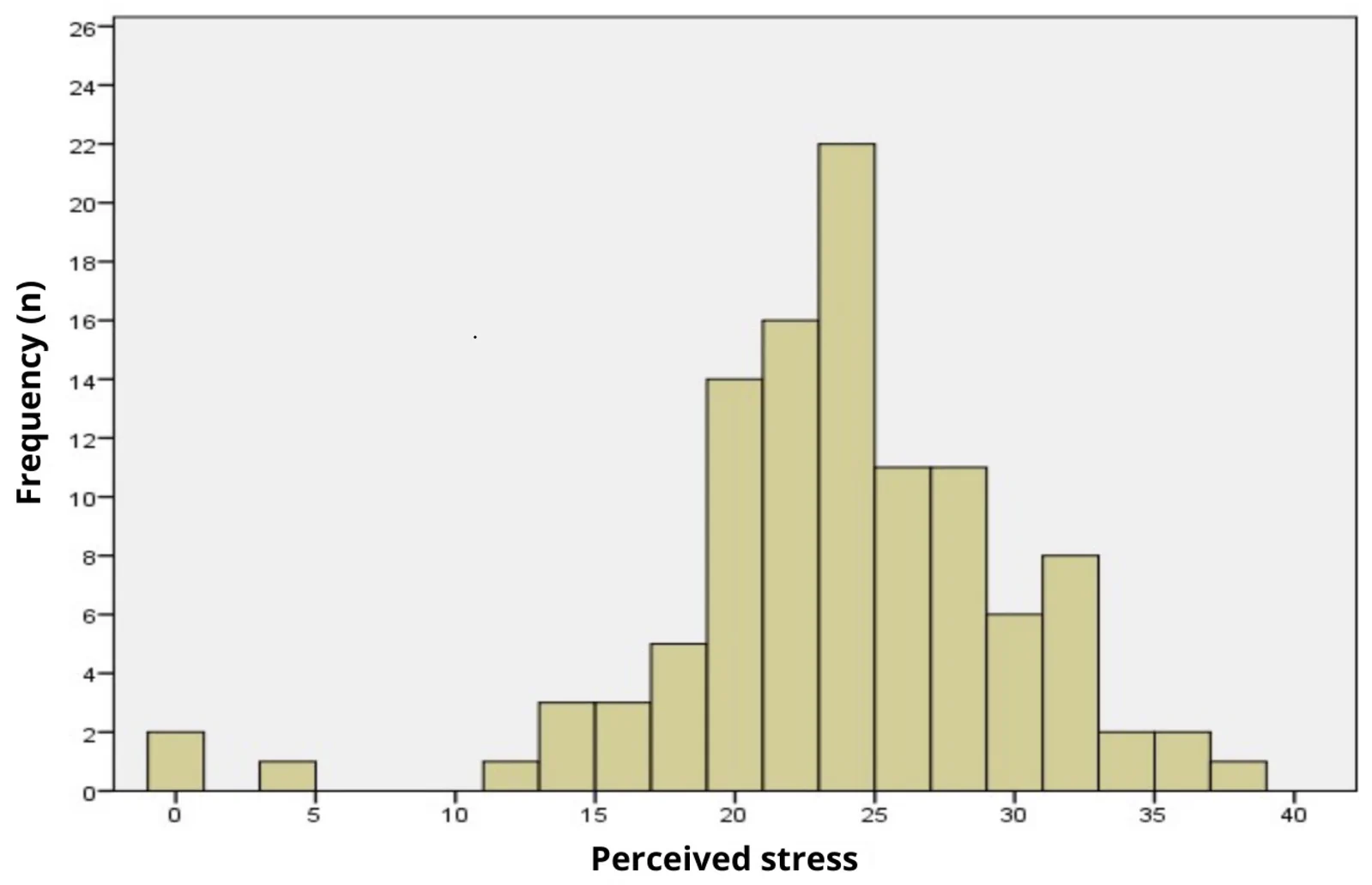
Perceived stress among study participants—raw PSS-10 scores.

This section presents the results obtained with the PSS-10 questionnaire, used to assess the level of perceived stress among respondents. The results are presented in Figure 1. 

The mean PSS-10 score was 23.14 [95% CI (21.94, 24.34)], with a standard deviation of 6.27. Half of the respondents scored 23 points or higher. The lowest score in the study group was 0 and the highest was 38. The Kolmogorov–Smirnov test showed a statistically significant deviation of the PSS-10 score distribution from a normal distribution (KS = 0.116; *p* = 0.001); the skewness and kurtosis coefficients (SKEW = −0.875; KURT = 2.931) indicated that this deviation was mainly attributable to leptokurtosis, i.e., a marked concentration of scores around the mean.

The Table 1 presents the results of the analysis of the association between respondents’ stress level and demographic variables.

No statistically significant association was found between respondents’ age and their level of perceived stress (rho = −0.037; *p* = 0.706).

The analyses showed a statistically significant, moderate association between place of residence and perceived stress. The highest level of stress was reported by respondents living in towns of up to 100,000 inhabitants, followed by those living in rural areas, with the lowest level among residents of cities with more than 100,000 inhabitants. Post hoc tests showed statistically significant differences in perceived stress between residents of towns up to 100,000 inhabitants and residents of cities above 100,000 inhabitants (*p* = 0.009), and between residents of cities above 100,000 inhabitants and residents of rural areas (*p* = 0.002). No significant difference was found between residents of small towns and rural areas.

### 3.3. Coping with Stress Among Oncology Patients

This Table 2 presents the strategies used by oncology patients to cope with stress, assessed with two instruments: the JSR (“How Do I Cope”) questionnaire, and the DS-14 (Type D Personality Scale). Associations between coping strategies and demographic variables were also examined.

The analyses revealed a marked elevation of Type D personality traits in the study group, affecting both negative affectivity and social inhibition, as summarized in Table 3 below.

The following Table 4 presents the results obtained with the JSR (“How Do I Cope”) questionnaire in comparison with the normative group.

The analysis showed that, compared with the normative group, respondents relied significantly less often on emotion-focused coping and, to a lesser extent, on seeking social support. No significant difference was found between respondents and the normative group in the frequency of active coping. The 95% confidence interval for this strategy [95% CI (1.87, 2.23)] fell within approximately half a standard deviation of the normative mean, suggesting an average level of reliance on active coping.

The tables below present the results of the analysis of associations between coping strategies and sociodemographic characteristics. Coping strategies and stress experience in relation to age are presented in Table 5.

No statistically significant associations were found between respondents’ age and their coping strategies. Coping strategies and stress experience in relation to sex are presented in Table 6.

No statistically significant associations were found between sex and respondents’ coping strategies. Coping strategies and stress experience in relation to education are presented in Table 7.

The analyses showed statistically significant, moderate associations between education and negative affectivity. The highest scores were obtained by respondents with secondary education, followed by those with higher, vocational, and primary education. Post hoc tests showed statistically significant differences in negative affectivity between respondents with primary education and those with vocational (*p* < 0.001), secondary (*p* < 0.001), and higher education (*p* < 0.001), and between respondents with vocational education and those with secondary (*p* < 0.001) and higher education (*p* = 0.024). No significant differences were found between the remaining groups.

The analyses also showed statistically significant, moderate associations between education and active coping. The highest scores were obtained by respondents with secondary education, followed by higher, vocational, and primary education. Respondents with primary education differed significantly from those with vocational (*p* < 0.001), secondary (*p* < 0.001), and higher education (*p* < 0.001); no significant differences were found between the remaining groups.

Statistically significant, moderate associations were also found between education and emotion-focused coping. The highest scores were obtained by respondents with secondary education, followed by higher, vocational, and primary education. Respondents with primary education differed significantly from those with vocational (*p* < 0.001), secondary (*p* < 0.001), and higher education (*p* < 0.001); respondents with vocational education differed significantly from those with secondary education (*p* = 0.038).

Statistically significant, moderate associations were found between education and seeking social support. The highest scores were obtained by respondents with secondary education, followed by higher, vocational, and primary education. Respondents with primary education differed significantly from those with vocational (*p* < 0.001), secondary (*p* < 0.001), and higher education (*p* < 0.001); respondents with vocational education differed significantly from those with secondary education (*p* = 0.020). No significant differences were found between the remaining groups.

Table 8 presents coping strategies and stress experience in relation to place of residence.

The analyses showed a statistically significant, moderate association between place of residence and seeking social support. The highest scores were obtained by residents of cities with more than 100,000 inhabitants, followed by residents of towns up to 100,000 inhabitants, with the lowest scores among rural residents. Post hoc tests showed statistically significant differences between residents of towns up to 100,000 inhabitants and rural residents (*p* < 0.001), and between residents of cities above 100,000 inhabitants and rural residents (*p* < 0.001).

## 4. Discussion

The findings confirm that oncological disease remains one of the most powerful health-related stressors, affecting both the psychological and social functioning of patients. The study group displayed a high level of perceived stress, significantly exceeding values observed in the normative population. These results are consistent with current directions in psycho-oncological research, which indicate that the psychological burden associated with a cancer diagnosis is not confined to the moment of diagnosis but persists throughout the entire treatment process and the subsequent follow-up period. Uncertainty regarding treatment efficacy, the possibility of disease recurrence, and concern about the future are among the most frequently reported sources of chronic stress in oncology patients.

Our findings of a high level of stress measured with the PSS-10 are consistent with the observations of Cooper et al. [10], who showed that a higher level of perceived stress is associated with poorer psychosocial functioning and lower quality of life among patients undergoing oncological treatment. These authors emphasized that protective factors such as mindfulness and social support significantly reduce stress levels while improving patients’ adaptive capacities. Our results further underscore the importance of routine stress assessment as an integral component of comprehensive oncological care.

An interesting aspect of this study is the absence of an association between the level of perceived stress and respondents’ age or sex. This finding is corroborated in some of the contemporary literature, which suggests that the diagnosis of cancer itself constitutes the dominant stressor, outweighing the influence of basic sociodemographic variables. Increasing emphasis is placed on the notion that individual psychological resources, prior life experience, psychological resilience, and the availability of social support are more important determinants of stress than age or sex. Consequently, patients from different age groups may experience comparable levels of stress, even though the way in which this stress is experienced and regulated may differ.

Noteworthy is the association found between place of residence and the severity of perceived stress. The highest level of stress was observed among residents of smaller towns, while the lowest was reported by residents of large urban agglomerations. This finding may reflect differences in access to highly specialized oncological care, psychological support, and organizations assisting patients and their families. The literature emphasizes that limited access to specialist services, greater distance from treatment centers, and fewer local support programs increase feelings of uncertainty and intensify stress among patients living outside large urban centers. This points to the need for further development of psycho-oncological services in smaller localities and for improved access to psychological consultations for patients treated away from major oncology centers.

Another important finding was the marked elevation of Type D personality traits, encompassing both negative affectivity and social inhibition. Type D personality is currently regarded as one of the most significant psychological risk factors for poorer adaptation to chronic illness. In oncology patients, a high level of negative affectivity has been shown to be associated with a higher incidence of depressive symptoms, increased anxiety, poorer quality of life, and reduced active participation in the treatment process [6,11]. Our findings are consistent with more recent longitudinal evidence showing that, in patients with breast cancer, both components of Type D personality—negative affectivity and social inhibition—are associated with higher anxiety, depression, and post-traumatic stress symptoms, as well as with maladaptive coping [11], and with reports indicating that patients with Type D personality traits more often interpret health-related situations as threatening, show a greater tendency toward catastrophizing, and experience greater difficulty adapting to chronic treatment [12].

Analysis of coping strategies showed that respondents displayed an average level of active coping, while relying less frequently than the normative population on emotion-focused coping and on seeking social support. This pattern may reflect gradual adaptation to the chronic treatment process, during which patients focus primarily on following medical recommendations and completing successive stages of treatment. At the same time, limited use of social support may constitute an unfavorable prognostic factor: current research consistently indicates that a high level of emotional support is associated with reduced stress, improved quality of life, and greater adherence to treatment recommendations. Contemporary psycho-oncology therefore emphasizes the need to actively involve patients’ families and multidisciplinary teams in the process of psychological support.

Our results also indicate that the level of education significantly differentiated both negative affectivity and preferred coping strategies. Respondents with secondary and higher education more often applied active coping strategies, more frequently sought social support, and showed greater readiness for constructive adjustment to illness. These findings are consistent with reports from contemporary authors, who emphasize that higher educational attainment is associated with greater health literacy, easier access to reliable medical information, and higher health-related competence. These factors favor the adoption of active coping strategies while reducing the sense of helplessness in the face of the treatment process.

It is noteworthy that, despite a high level of perceived stress, respondents did not display a significantly lower level of active coping than the reference population. This may indicate gradual adaptation to chronic illness and mobilization of psychological resources during successive stages of treatment. Contemporary models of adaptation to cancer assume that effective coping does not depend solely on the intensity of stress but, above all, on psychological flexibility and the capacity to modify coping strategies as the clinical situation evolves. Patients can therefore maintain a high level of stress while simultaneously preserving active, treatment-supportive behaviors.

Our finding of relatively infrequent use of social support warrants particular attention from a clinical perspective. Numerous studies show that emotional support from family, medical staff, and self-help groups is one of the most important factors protecting against chronic psychological distress in oncology patients. Well-developed support networks favorably influence not only psychological well-being but also adherence to treatment, treatment satisfaction, and quality of life. Accordingly, it is increasingly recommended that routine assessment of psychosocial needs be incorporated as an integral element of oncological care.

These results are also consistent with the current model of patient-centered care, according to which effective treatment of cancer should encompass not only antineoplastic therapy but also systematic assessment of psychological well-being and implementation of appropriate psycho-oncological interventions. In recent years, particular importance has been attached to programs based on cognitive behavioral therapy, mindfulness training (Mindfulness-Based Cognitive Therapy, Mindfulness-Based Stress Reduction), acceptance and commitment therapy, and interventions strengthening psychological resilience. These methods have been shown to significantly reduce anxiety and stress, improve quality of life, and enhance the adaptive capacities of oncology patients.

It should be emphasized that contemporary psycho-oncology increasingly focuses on identifying patients at elevated risk of adjustment disorders, particularly those presenting high negative affectivity, a limited social support network, and difficulty with active coping. Early identification of such patients enables the implementation of preventive measures before the onset of fully symptomatic anxiety or depressive disorders, which may favorably affect both the course of treatment and patients’ quality of life.

These findings also have important practical implications for nursing staff and the entire therapeutic team. Nurses, who remain in the closest contact with patients during hospitalization and outpatient treatment, play a key role in identifying symptoms of severe stress and referring patients to specialist psychological support. Regular assessment of stress levels, monitoring of coping strategies, and patient education regarding available forms of support should constitute an integral part of standard oncological care. This approach is consistent with the recommendations of contemporary organizations involved in psycho-oncology, which advocate treating distress as one of the fundamental parameters requiring systematic assessment during cancer treatment [10,13,14,15], a conclusion reinforced by a recent review of distress-screening practice specifically addressing implications for oncology nursing [16].

### 4.1. Strengths of the Study

Among the principal strengths of this study is the use of standardized, validated psychometric instruments enabling a comprehensive assessment of stress level, Type D personality traits, and coping strategies. An additional strength is the simultaneous analysis of associations between psychological and sociodemographic variables, allowing for a more multidimensional interpretation of the results. The inclusion of patients from several oncological departments further enhances the clinical relevance of the observations.

### 4.2. Limitations and Future Directions

Several limitations should be considered when interpreting these results. The cross-sectional design precluded assessment of changes in stress level and coping strategies across successive stages of treatment and post-treatment follow-up. The sample size and the single-center design limit the generalizability of the findings to the broader population of oncology patients. Future prospective, multicenter studies involving larger samples, incorporating clinical disease characteristics, and evaluating the effectiveness of individualized psycho-oncological interventions are warranted. Such studies would help to better identify factors that promote effective adaptation and to develop support models tailored to the needs of different patient groups. A major limitation is the absence of clinical characterization of the study sample: no data were collected on cancer diagnosis or primary tumor site, disease stage, presence of metastatic disease, time elapsed since diagnosis, current treatment status, or the specific therapeutic regimen received (e.g., surgery, chemotherapy, radiotherapy, or a combination thereof). These variables are well-established determinants of psychological distress and coping responses and may have confounded the sociodemographic associations reported here; for example, patients recently diagnosed or undergoing active chemotherapy may experience different stress profiles than those in surveillance after successful treatment. This omission reflects the original survey design, which prioritized full anonymity of participants and did not extract data from medical records, and it considerably limits the clinical interpretation and generalizability of the present findings. This limitation should be regarded as a priority for future research.

A further limitation concerns the recruitment procedure and the extended data-collection period. Sampling was consecutive within the participating departments rather than based on a formal randomized design, which may have introduced selection bias. The data-collection period extended from December 2021 to April 2025. Changes in oncological treatment protocols, hospital organization, or psychosocial care practices over this period cannot be excluded and may have introduced additional heterogeneity into the cross-sectional sample. This possibility should be considered when interpreting the results and should be explicitly discussed.

Future prospective, multicenter studies involving larger samples should incorporate detailed clinical disease characteristics (diagnosis, stage, metastatic status, time since diagnosis, and treatment modality) alongside psychometric assessment, and should evaluate the effectiveness of individualized psycho-oncological interventions. Such studies would help to better identify factors that promote effective adaptation, disentangle the contribution of clinical from sociodemographic determinants of distress, and support the development of support models tailored to the needs of different patient groups.

## 5. Conclusions

As this was a single-center, cross-sectional study with a modest sample size and no clinical disease characterization, the conclusions below should be interpreted as hypothesis-generating observations linked directly to the associations found in this sample, rather than as generalizable or causal claims; broader generalization would require confirmation in larger, multicenter, prospective studies.

The high intensity of stress observed in this sample of oncology patients suggests a need for routine assessment of psychological distress at every stage of diagnosis, treatment, and post-treatment follow-up, pending confirmation in larger studies. Early identification of patients with elevated stress may enable the implementation of appropriate psycho-oncological interventions, which may improve therapeutic cooperation and quality of life.

The absence of an association between stress level and age or sex in this sample suggests that psychological support could reasonably be offered to all oncology patients, regardless of their demographic characteristics, rather than being restricted to specific age or sex groups.

The influence of education on coping strategies points to the need for individualized health education. Patients with lower educational attainment may require more intensive educational support, simplified communication of information, and more frequent reinforcement of strategies for coping with illness and stress.

Patients’ limited use of social support indicates the need to actively involve family members and to refer patients to a psychologist, psycho-oncologist, or support group. The therapeutic team should proactively initiate measures to strengthen the patient’s social support network rather than waiting for the patient to request such support.

The marked elevation of Type D personality traits suggests the need to identify patients particularly susceptible to adjustment difficulties. Incorporating brief screening tools into clinical practice may facilitate the identification of patients requiring more intensive psychological care and systematic monitoring of their mental state.

The maintenance of an average level of active coping despite a high level of stress indicates that medical staff should reinforce adaptive coping strategies through education, motivation, and the strengthening of patients’ sense of agency. Such measures may increase patient engagement in the treatment process and adherence to therapeutic recommendations.

The higher level of stress observed among patients living outside large urban centers points to the need to improve the availability of psycho-oncological services in smaller localities. The development of psychological consultations, telemedicine, and local support programs may help reduce inequalities in care for oncology patients.

Comprehensive oncological care could benefit from systematic monitoring of psychological well-being as an integral part of the therapeutic process. Regular collaboration among physicians, nurses, psychologists, and psycho-oncologists may support the early identification of emotional problems and could improve the effectiveness of treatment and patients’ quality of life.

## Figures and Tables

**Table 1:** Perceived stress in relation to sociodemographic variables.

Demographic Variable	Level of Variable	M	SD	*n*	Mr	H	df	*p*	ε^2^
Sex	Female	23.78	5.15	51	54.03	0.000	1	0.993	0.000
	Male	22.96	6.51	56	53.97				
Education	Primary	17.88	14.53	8	48.81	1.038	3	0.792	0.010
	Vocational	22.74	3.80	19	51.18				
	Secondary	23.38	4.92	42	53.70				
	Higher	24.15	5.65	39	58.14				
Place of residence	Town ≤ 100,000 inhabitants	25.28	5.06	18	64.17	8.518	2	0.014	0.081
	City > 100,000 inhabitants	19.21	7.23	65	44.32				
	Rural area	21.46	5.54	25	52.34				

M—mean; SD—standard deviation; *n*—number of observations; Mr—mean rank; H—Kruskal–Wallis test statistic; df—degrees of freedom; *p*—test probability; ε^2^—epsilon-squared.

**Table 2:** Descriptive and distributional statistics of the DS-14 and JSR subscales.

Scale	M	95% CI	SD	Median	Min	Max	KS	*p*	SKEW	KURT
Negative affectivity	16.53	15.63–17.43	4.71	17.00	0	27	0.141	<0.001	−0.894	1.747
Social inhibition	13.56	12.62–14.51	4.94	15.00	0	25	0.140	<0.001	−0.670	0.471
Active coping	2.05	1.87–2.23	0.95	2.00	0	4	0.124	<0.001	−0.186	−0.887
Emotion-focused coping	1.70	1.52–1.88	0.94	1.67	0	4	0.116	0.001	0.285	−0.259
Seeking social support	1.72	1.52–1.92	1.04	1.50	0	4	0.143	<0.001	0.401	−0.779

M—mean; CI—confidence interval; SD—standard deviation; KS—Kolmogorov–Smirnov test statistic; *p*—test probability; SKEW—skewness coefficient; KURT—kurtosis coefficient. All five distributions deviated significantly from normality (*p* ≤ 0.001), which is why non-parametric methods were used for all within-sample association analyses involving these scales (see Section 2.3).

**Table 3:** Type D personality traits in the study group compared with the normative sample.

Type D Personality Trait	Normative Group M	Normative Group SD	Study Group M	Study Group SD	t	df	*p*	d
Negative affectivity	10.96	6.01	16.53	4.71	12.287	107	<0.001	0.926
Social inhibition	7.47	5.53	13.56	4.94	12.818	107	<0.001	1.102

M—mean; SD—standard deviation; *p*—test probability; t—Student’s *t*-test statistic; df—degrees of freedom; d—Cohen’s d effect size.

**Table 4:** Comparison of coping strategies used by respondents with the highest normative age-group.

Coping Strategy	Normative Group M	Normative Group SD	Study Group M	Study Group SD	t	df	*p*	d
Active coping	2.02	0.88	2.05	0.95	0.289	107	0.773	0.030
Emotion-focused coping	2.75	0.88	1.70	0.94	−11.581	107	0.000	1.192
Seeking social support	2.02	1.02	1.72	1.04	−3.028	107	0.003	0.298

M—mean; SD—standard deviation; *p*—test probability; t—Student’s *t*-test statistic; df—degrees of freedom; d—Cohen’s d effect size.

**Table 5:** Coping strategies and stress experience in relation to age.

Coping Strategy/Stress Experience	rho	*p*
Negative affectivity	0.015	0.880
Social inhibition	0.063	0.518
Active coping	0.143	0.140
Emotion-focused coping	0.007	0.943
Seeking social support	0.062	0.526

rho—Spearman’s rho correlation coefficient; *p*—test probability.

**Table 6:** Coping strategies and stress experience in relation to sex.

Coping Strategy	Sex	M	SD	*n*	Mr	H	df	*p*	ε^2^
Negative affectivity	Female	16.98	3.71	51	54.24	0.006	1	0.940	0.000
	Male	16.39	5.10	56	53.79				
Social inhibition	Female	13.61	4.41	51	52.56	0.212	1	0.645	0.002
	Male	13.66	5.36	56	55.31				
Active coping	Female	2.14	0.94	51	56.38	0.582	1	0.446	0.006
	Male	1.99	0.92	56	51.83				
Emotion-focused coping	Female	1.84	0.88	51	57.44	1.217	1	0.270	0.012
	Male	1.61	0.97	56	50.87				
Seeking social support	Female	1.91	0.95	51	60.04	3.735	1	0.053	0.036
	Male	1.56	1.11	56	48.50				

*n*—number of observations; M—mean; SD—standard deviation; Mr—mean rank; H—Kruskal–Wallis test statistic; *p*—test probability; df—degrees of freedom; ε^2^—epsilon-squared.

**Table 7:** Coping strategies and stress experience in relation to education.

Coping Strategy	Education	M	SD	*n*	Mr	H	df	*p*	ε^2^
Negative affectivity	Primary	11.13	7.70	8	32.31	9.027	3	0.029	0.086
	Vocational	15.37	3.88	19	44.39				
	Secondary	17.83	3.99	42	62.81				
	Higher	16.79	4.29	39	55.03				
Social inhibition	Primary	11.38	5.10	8	40.25	2.879	3	0.411	0.027
	Vocational	13.11	4.07	19	50.76				
	Secondary	14.40	4.44	42	59.15				
	Higher	13.33	5.74	39	54.23				
Active coping	Primary	1.00	0.98	8	23.44	9.236	3	0.026	0.088
	Vocational	2.02	0.89	19	52.05				
	Secondary	2.17	0.93	42	58.80				
	Higher	2.14	0.89	39	57.44				
Emotion-focused coping	Primary	0.71	0.74	8	23.25	10.134	3	0.017	0.096
	Vocational	1.63	1.04	19	50.05				
	Secondary	1.83	0.71	42	60.23				
	Higher	1.80	1.05	39	56.91				
Seeking social support	Primary	1.00	1.15	8	29.94	7.968	3	0.047	0.076
	Vocational	1.60	0.96	19	50.92				
	Secondary	1.96	1.01	42	62.30				
	Higher	1.66	1.05	39	52.88				

*n*—number of observations; M—mean; SD—standard deviation; Mr—mean rank; H—Kruskal–Wallis test statistic; *p*—test probability; df—degrees of freedom; ε^2^—epsilon-squared.

**Table 8:** Coping strategies and stress experience in relation to place of residence.

Coping Strategy	Place of Residence	M	SD	*n*	Mr	H	df	*p*	ε^2^
Negative affectivity	Town ≤ 100,000	16.89	4.10	18	55.92	0.176	2	0.916	0.002
	City > 100,000	16.49	4.45	65	53.48				
	Rural area	16.36	5.83	25	56.14				
Social inhibition	Town ≤ 100,000	13.50	3.94	18	48.61	2.066	2	0.356	0.020
	City > 100,000	14.02	4.91	65	58.00				
	Rural area	12.44	5.64	25	49.64				
Active coping	Town ≤ 100,000	1.87	0.70	18	47.56	4.307	2	0.116	0.041
	City > 100,000	2.20	0.93	65	59.55				
	Rural area	1.77	1.10	25	46.38				
Emotion-focused coping	Town ≤ 100,000	1.44	0.56	18	46.64	1.413	2	0.493	0.013
	City > 100,000	1.74	0.91	65	56.43				
	Rural area	1.79	1.21	25	55.14				
Seeking social support	Town ≤ 100,000	1.80	0.87	18	57.36	10.030	2	0.007	0.095
	City > 100,000	1.90	1.07	65	60.32				
	Rural area	1.19	0.94	25	37.32				

*n*—number of observations; M—mean; SD—standard deviation; Mr—mean rank; H—Kruskal–Wallis test statistic; *p*—test probability; df—degrees of freedom; ε^2^—epsilon-squared.

## Data Availability

The data presented in this study are available on request from the corresponding author.
